# A Rare Case of Ectopic Adrenocorticotropic Hormone Syndrome (EAS) in an Adolescent Girl With a Thymic Neuroendocrine Tumour

**DOI:** 10.7759/cureus.66615

**Published:** 2024-08-10

**Authors:** Magdy F Ahmed, Shahida Ahmed, Asmaa Abdussalam, Fatemah Alhusaini

**Affiliations:** 1 Paediatrics, Southend University Hospital, Southend, GBR; 2 Paediatrics, Basildon & Thurrock University Hospital, Basildon, GBR; 3 Paediatrics and Child Health, Broomfield Hospital, Chelmsford, GBR; 4 Paediatrics, Great Ormond Hospital, London, GBR

**Keywords:** gallium-dota-tate pet scan, primary neuroendocrine tumour, cushing syndrome, neuroendocrine tumour, ectopic acth syndrome

## Abstract

In adults, Cushing's syndrome (CS) caused by tumours that produce adrenocorticotropic hormone (ACTH) outside of the pituitary gland is quite prevalent. However, it is uncommon in children and teenagers. Ectopic ACTH syndrome (EAS) is a rare occurrence in young children, accounting for less than 1% of cases. Nevertheless, when it does occur, it tends to be a severe condition due to the profound hypercortisolism that can occur independently of the tumour. Consequently, EAS should be regarded as a critical endocrine emergency, necessitating prompt action in terms of diagnostic procedures and therapeutic interventions. A 14-year-old White female from the United Kingdom (UK) presented with a two-week history of hands and feet swelling, accompanied by a non-itchy rash on the face, back, and chest for one week. Initial investigations revealed hypokalemic alkalosis, hyperglycaemia, elevated serum and 24-hour urinary cortisol, and high ACTH level. Further investigations confirmed EAS, and to find out the primary tumour location, a contrast-enhanced thoracic CT scan was done and identified a 5x3 cm mass with enhanced thymic shadow. Increased radioactivity uptake in the left upper chest along with widespread GAD-avid metastatic disease unveiled by Gallium-DOTA-TATE PET scan. An abdominal MRI, detecting multiple liver deposits, prompted a liver biopsy, revealing a malignant tumour with neuroendocrine differentiation. The patient was diagnosed with EAS with a primary neuroendocrine tumour of the thymus and metastasis. This case underscores the significance of considering EAS in patients with a diagnosis of CS, especially in young individuals with no known risk factors.

## Introduction

In adults, Cushing's syndrome (CS) caused by tumours that produce adrenocorticotropic hormone (ACTH) outside of the pituitary gland is quite prevalent. However, it is uncommon in children and teenagers. Ectopic ACTH syndrome (EAS) is a rare occurrence in young children, accounting for less than 1% of cases. Nevertheless, when it does occur, it tends to be a severe condition due to the profound hypercortisolism that can occur independently of the tumour. Consequently, EAS should be regarded as a critical endocrine emergency, necessitating prompt diagnostic and therapeutic interventions [1].

The management of patients with CS and neuroendocrine tumours (NET) requires a multifaceted approach, encompassing expertise in both diagnosis and treatment. The dual skills necessary include addressing the consequences of excess cortisol, managing the non-specific manifestations of hypercortisolism, and providing appropriate preventive and curative treatments.

This comprehensive approach takes into account various factors, such as the severity of hypercortisolism, the overall health of the patient, any associated conditions, and the specific tumour status, which can range from resectable ACTH-secreting tumours to non-resectable metastatic endocrine tumours or occult tumours. Therefore, the management strategies need to be tailored accordingly to suit the individual circumstances [2].

The optimal treatment approach involves the complete removal of the ACTH-secreting tumour, either through prompt surgical excision or following preparatory measures using medications to lower cortisol levels. However, in situations where complete excision is not feasible, the therapeutic plan should be carefully deliberated by an experienced multidisciplinary team, considering the unique circumstances of each patient. This personalized perspective may entail a combination of different pharmacological treatments, bilateral adrenalectomy (surgical removal of both adrenal glands), and non-specific tumoural interventions [2].

Here, we report a case of an early adolescent girl with EAS associated with a thymic neuroendocrine tumour with metastasis.

## Case presentation

A previously healthy White early adolescent girl presented with a two-week history of swelling in her hands and feet and a one-week history of a non-pruritic rash on her face, chest, and back. Her past medical history was unremarkable. She denied the use of steroid medications or any drug allergies. She had started menarche at the age of 11 years, with irregular periods lasting seven days, menorrhagia, and dysmenorrhea. Her maternal grandmother had a history of type 2 diabetes mellitus and hypertension, but there was no family history of endocrine disorders. The patient's weight was 75 kg, with a height of 160 cm (50th centile) and a BMI of 99th centile.

Physical examination showed new-onset hirsutism, acneiform eruptions over the face and back, swelling of hands and feet, and striae on the abdomen. She had hyperglycaemia and hypokalaemia, so she was managed at the local hospital with intravenous fluids, potassium supplements, an insulin sliding scale, and antihypertensive medications to control her high blood pressure. Further blood results confirmed CS due to ectopic ACTH secretion. Consequently, she was transferred to the tertiary hospital for ongoing endocrine care, where she was started on treatment with metyrapone. Further work-up confirmed ectopic adrenocorticotropic hormone syndrome due to a thymic neuroendocrine tumour with metastases. The patient was jointly managed by the local, tertiary endocrine, and oncology teams.

Investigations

The initial blood results showed a pH of 7.53, HCO_3_ of 33.9, Na of 142 mmol/L, and K of 2.6 mmol/L. Owing to the recent onset of acneiform rash, hypertension, and results of initial blood tests, 8 AM paired serum ACTH and cortisol tests were requested. Serum cortisol came back to be significantly elevated, 810 g/dL (N: 5-25 g/dL), and high ACTH levels, 328 pg/mL (N: < 50 pg/mL)).

To confirm the hypercortisolism, overnight dexamethasone suppression was done, which revealed a non-suppressed cortisol. Twenty-four hours of urinary free cortisol (UFC) was very high, 81,146 nmol/day (normal < 200). A high-dose dexamethasone suppression test revealed a non-suppressed cortisol (> 1,655 mcg/dL (N: < 1.8 mcg/dL)). The results of further blood tests are shown in Table 1. 

**Table 1:** Further blood tests

Test	Results	Reference range
Serum androstenedione	41.2 nmol /L	2-5.4 nmol/L
Serumdehydro-epiandrostenedione	15.7 umol/L	1.6-7.8 umol/L
Testosterone level	6.3 nmol/L	2.4 nmol/L
Prolactin	234 mU/L	71-566
FSH	0.6 IU/L	0.3-10 IU/L
LH	0.2 U/L	0.03-3.9 U/L
17 OH Progesterone	8.3 nmol/L	<18
Oestradiol	59 pmol/L	45.4-1461 pmol/L
Urine VMA/HVA, catecholamines and metanephrines.	Normal	
bHCG	<2.391 U/L	
CEA	3 ug/L	
Chromogranin A	25 pmol/L,	
Glucagon	33 pmol/L	
MEN, CDC73, CDKN1B/RET mutation	Negative	

**Figure 1:**
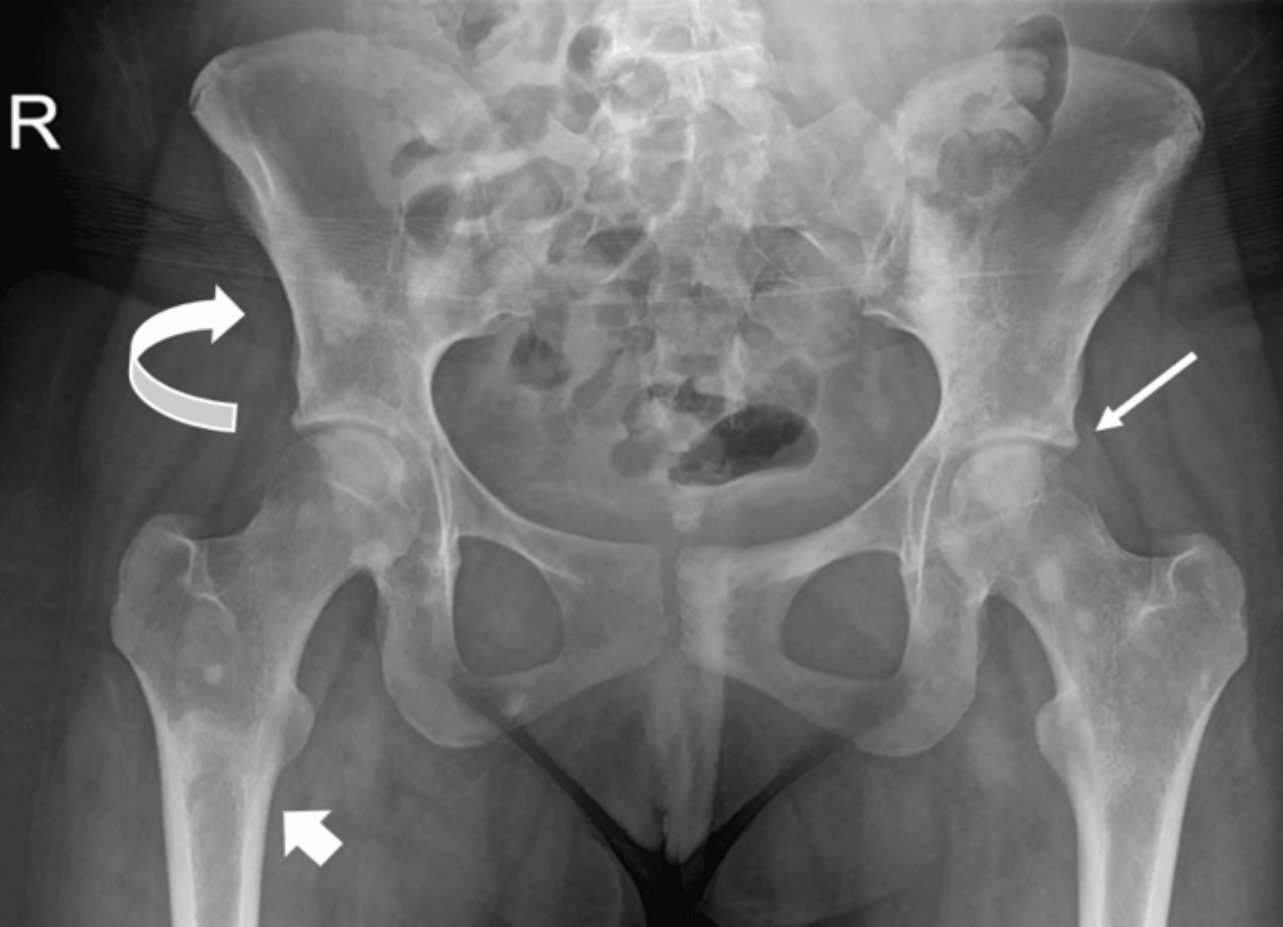
Plain X-ray of the bony pelvis and upper femoral bones showing multiple sclerotic areas. Right iliac bone lesion (curved arrow), left femoral head (small white arrow), and right upper femoral shaft( subtrochanteric region, white arrow)

In terms of imaging, an initial chest X-ray showed bilateral pleural and pericardial effusion. A repeated image was done in view of frequent episodes of desaturation, showing new subsegmental left lower lobe atelectasis with a little fluid in the pleural spaces bilaterally. In addition, there was developing upper lobe consolidation on the right and subsegmental atelectasis in the right middle lobe. Multiple sclerotic bone lesions in the pelvis and proximal femora were noted on the hip X-ray (Figure 1).

CT thorax with contrast revealed enhancing soft tissue seen in the superior mediastinum, measuring 5x3 cm. The configuration suggested thymic infiltration lying in the aortopulmonary window. A small pericardial effusion, measuring up to 14 mm in depth, bilateral pleural effusions, some compressive atelectasis within adjacent lung parenchyma next to the effusions, and multiple sclerotic foci seen within the imaged skeleton were noted. Thyroid ultrasound showed a normal thyroid gland and parathyroid adenoma (3x5 mm), and bone profile and parathyroid hormone were within normal levels, suggesting that this is not functioning.

**Figure 2:**
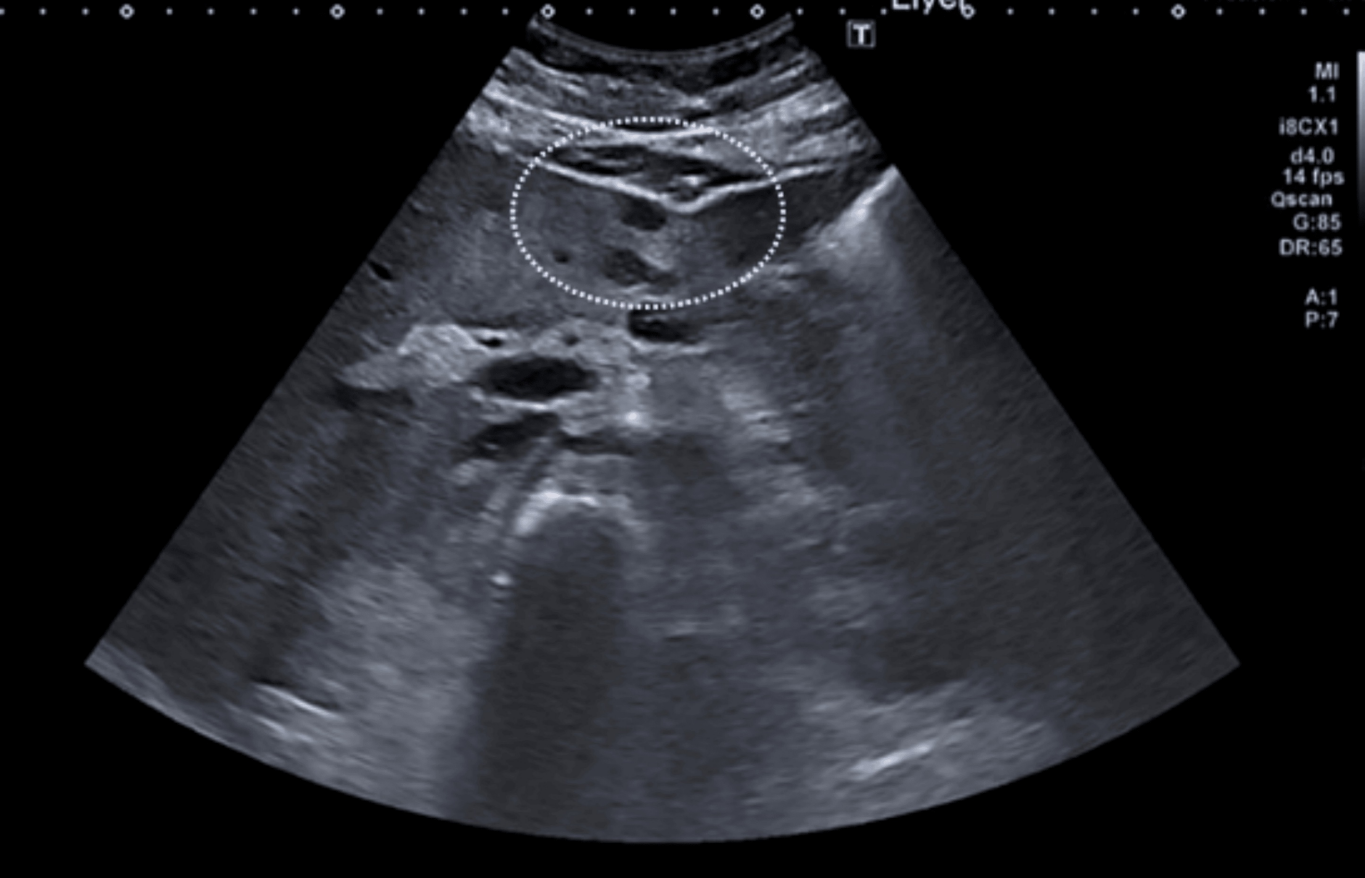
US of the abdomen. The liver shows multiple small hypoechoic lesions, dashed circle)

A 7.3 mm filling defect in the pituitary in keeping with a pituitary micro adenoma was noted on pituitary MRI. Abdominal US showed enlarged liver with multiple hypoechoic lesions (Figure 2).

Innumerable liver and bony metastatic deposits, as well as enlarged adrenal glands with no discrete nodules, were noted on abdominal MRI with contrast. Other findings include an enlarged pre-caval lymph node immediately posterior to the pancreatic head measuring 15 mm in the short axis and a few abnormal porta hepatis nodes, the largest measuring 12 mm in the short axis. No definitive primary lesion or evidence of mesenteric abnormality to suggest a gastrointestinal carcinoid.

**Figure 3:**
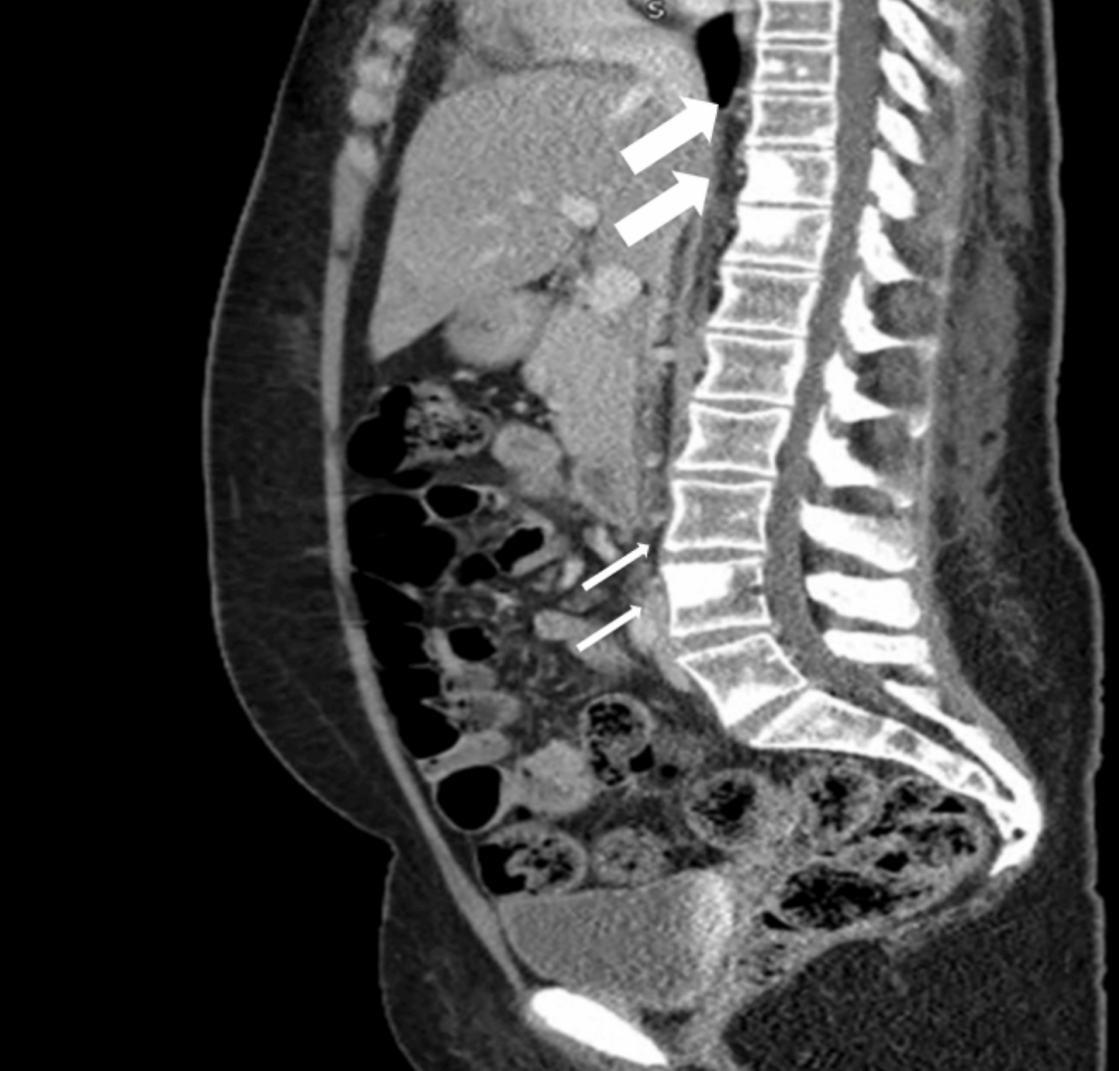
CT abdomen and pelvis (sagittal reconstruction) showing multiple scattered sclerotic lesions involving the lumbar spine (small and large arrows)

CT abdomen showed a number of small lesions throughout the liver and Innumerable sclerotic lesions throughout the imaged skeleton, and the most diffusely involved bones are the superior left pubic ramus (which demonstrates some ground-glass change within the sclerotic lesions) and the left ilium, and several large lesions are seen throughout the imaged spine, as shown in Figure 3.

A Gallium-DOTA-TATE (GAD-PET) scan demonstrated multiple widespread DOTATATE avid metastases within the liver; intrathoracic and intra-abdominal lymph nodes; soft tissue of the breast and skeleton, including the imaged skull base; left aspect of the sphenoid body; and multiple vertebral bodies, including the anterior aspect of C3, the left scapula, throughout the pelvis, and within the imaged proximal femora. 

In view of the abdominal MRI findings and to rule out a neuroendocrine tumour, histopathology after liver ultrasound-guided biopsies was done and demonstrated a malignant tumour, showing neuroendocrine differentiation. A panel of antibodies shows the following results: The tumour cells show diffuse expression of CD56, synaptophysin and chromogranin, and weak membranous staining with beta-catenin and cytokeratin. Cytokeratin also highlights numerous residual bile ducts. CD45 highlights scattered lymphocytes but is negative in the tumour. CD99, BCOR, inhibin, S100, PHOX2b, and glypican are all negative. Integrase interactor 1 (INI1) expression is retained. CD31 and CD34 demonstrate the vascular network but are negative in the tumour.

Treatment

The patient's initial care at the local hospital addressed hyperglycaemia and hypokalaemia. She received insulin on a sliding scale and was placed on a full IV maintenance fluid regimen with potassium chloride (20 mmol/L).

Subsequent potassium measurements consistently indicated low levels, so oral potassium supplementation was added. The total IV potassium chloride she received was 240 mmol and the oral KCL was 384 mmol. After two days of hospitalization, her potassium level was normalized (4 mmol). To manage her high blood pressure, spironolactone and frusemide (25 mg and 20 mg once a day, respectively) were incorporated into her medication regimen. Furthermore, she began a twice-daily subcutaneous low-molecular-weight heparin regimen with a 20 mg dose twice a day.

She was transferred to the tertiary hospital where she was initiated on metyrapone by the endocrine team, beginning with a dose of 200 mg three times a day. The dosage was carefully tailored, guided by sequential serum cortisol measurements, and the most recent dose of metyrapone has been escalated to 2,000 mg three times a day. Notably, surgical resection was deemed unfeasible due to the extensive metastasis.

During her stay under the care of the tertiary endocrine team, she was complaining of significant pain, particularly in her spine, likely secondary to metastases.

Her pain was controlled with gabapentin 300 mg three times a day, morphine sulphate 15 mg twice a day for long-acting control, and morphine 5 mg every four hours for breakthrough pain. She was given a pamidronate infusion for bone pain and continued monthly on it. In view of her low calcium level, she remained on calcium carbonate 2,000 mg three times a day for one week post pamidronate infusion.

She had a peripherally inserted central catheter (PICC) associated basilic vein non-occlusive thrombus for which enoxaparin was commenced and was switched to oral apixaban 5 mg twice a day for two weeks and then reduced to 2.5 mg twice a day. It was discontinued after the removal of the PICC line.

Following the transfer of care to the tertiary oncology team, the patient was initiated on a monthly regimen of lanreotide injections at a dosage of 90 mg.

Outcome and follow-up

Prior to discharge from the endocrine team care, she was reviewed by the endocrine clinical nurse specialist for teaching and emergency hydrocortisone injection training if unwell with double the usual doses of hydrocortisone and an additional 4 am dose to be added for 24-48 hours. The plan was to attend the local hospital to repeat blood, including full blood count, biochemistry, and regular blood pressure check, as well as follow up with the endocrine team regarding cortisol monitoring.

Following the oncology multidisciplinary team, both patient and parent understand that disease progression is expected as the current treatment consisting of monthly lanreotide and bisphosphonate infusions has been given to control symptoms. The patient has been attending school on a regular basis with no longer experiencing significant pain as she did at diagnosis despite extensive and widespread disease burden. They understand this treatment is not being given with curative intent.

An application for compassionate use of molecular radioisotope therapy with lutetium DOTATATE has been made and is still awaiting the outcome of this application. The patient and parents understand that, should this application be successful, treatment would be given with palliative intent.

## Discussion

We report a case of an adolescent girl who presented with rapid-onset symptoms suggestive of hypercortisolemia. The patient was diagnosed with EAS with a primary neuroendocrine tumor (NET) of the thymus with metastasis.

EAS arises due to dysregulated expression and release of ACTH by NETs found in various locations, exhibiting diverse levels of histological differentiation and aggressiveness. Consequently, these tumors typically trigger excessive cortisol secretion by the adrenal cortex.

The most frequent occurrence of EAS is observed in association with NET, specifically small-cell lung cancer (accounting for approximately 50% of cases), carcinoid syndrome, and medullary carcinoma of the thyroid [3,4]. There have also been documented cases of EAS in individuals with adenocarcinoma of the lung [5], breast, prostate, and pancreas [6,7].

A diagnosis of EAS is established when patients exhibit clinical manifestations indicative of excessive corticosteroid production and fulfil at least two of the following criteria: spontaneous hypokalemia (potassium of < 3.2 mmol/L), elevated plasma cortisol level (> 660 nmol/L) lacking diurnal variation and/or unresponsive to dexamethasone suppression, elevated plasma ACTH level (> 22 pmol/L), and/or increased 24-hour urinary free cortisol level (> 400 nmol/day). These specific criteria aid in the identification and diagnosis of EAS in affected individuals [8].

To differentiate between EAS and Cushing's disease, inferior petrosal sinus sampling can be employed. This invasive procedure involves sampling ACTH levels from the inferior petrosal sinuses, aiding in the determination of the source of excess ACTH production.

By comparing the ACTH levels obtained through this technique, it becomes possible to differentiate between EAS, which originates from a non-pituitary tumor and Cushing's disease, which is caused by a pituitary adenoma [9].

Thymic NETs are uncommon, with an estimated yearly occurrence of 0.07-0.18 cases per million individuals. The incidence tends to be higher among Caucasians and males. However, thymic NETs that produce ACTH do not display a gender preference and are typically diagnosed during early adulthood, specifically between the ages of 21 and 35. The prevalence of thymic NET in patients with multiple endocrine neoplasia type 1 (MEN1) varies between 2% and 8.2% in different research studies [10,11].

Thymic carcinoids are linked to an increased risk of mortality, with a hazard ratio of 4.64 and a 95% confidence interval of 1.73 to 12.41. Reports indicate that the median survival rate after the diagnosis of a thymic tumour is approximately 9.5 years, with a 10-year probability of survival estimated at 36.1% (ranging from 11.5% to 62%) [10,12]. The poorer prognosis can be attributed to the presence of advanced disease upon presentation, as most patients do not exhibit the typical features of carcinoid syndrome.

In patients with MEN1, carcinoid tumours often remain silent and devoid of clinical symptoms until reaching an advanced stage with malignant characteristics. Since thymic tumours associated with MEN can exhibit aggressive behaviour and manifest at any age, early detection becomes crucial. It is important to note that no consistent hormonal or biochemical abnormalities are observed in individuals affected by these carcinoid tumours. As a result, screening primarily relies on radiological imaging techniques, and regular imaging screenings can potentially enhance survival rates by facilitating early detection [13]. 

In clinical practice, genetic mutational analysis for MEN1 has several important purposes, including confirmation of diagnosis, identification of positive kindreds to facilitate early, regular screening, and identification of 50% of family members who do not have the germline mutation to allay any anxiety over future tumour burden for them and their progeny [13].

Mutation testing for MEN1 should be considered for patients who have been diagnosed with two or more tumours associated with MEN1 and patients who exhibit clinical indications of MEN syndrome, such as the presence of multiple parathyroid adenomas before the age of 45 and recurrent hyperparathyroidism or multiple enter pancreatic NETs at any age and first-degree relatives of known MEN1 mutation carriers, irrespective of whether they display symptoms.

It is worth noting that approximately 5-10% of patients with clinical features of MEN1 may not possess an identifiable mutation in the MEN1 gene. In such cases, an alternative genetic diagnosis should be pursued based on clinical suspicion. Possible alternative genetic causes may involve CDKN1B, CDKN1A (p21), CDKN2B (p15), CDKN2C (p18), HRPT2/CDC73, CASR, and AlP genes.

The diagnosis of EAS is generally not difficult; however, determining the exact origin of the ACTH-secreting tumour poses a challenge, particularly in the early stages. The key diagnostic features are the presence of a significant amount of ACTH in a tumour arising from tissues other than the pituitary gland, and the patient exhibits elevated levels of plasma ACTH and cortisol and failure of cortisol suppression after high dexamethasone administration or to rise following metyrapone testing. While these criteria are essential for diagnosing EAS, accurately localizing the tumour responsible for ACTH production remains a challenge, especially in the initial stages.

Accurate localization of EAS necessitates the utilization of various imaging techniques, predominantly computed tomography (CT), magnetic resonance imaging (MRI), somatostatin-receptor-based diagnostic imaging, and positron emission tomography (PET). CT and MRI scans are routinely employed for the diagnosis of EAS, providing valuable insights into the affected areas. Studies have shown that CT sensitivity (53%) is higher than that of MRI (37%) [14].

The preferred and most reliable method of functional imaging for NETs is somatostatin receptor scintigraphy, which specifically targets somatostatin receptor type 2 (SSTR2). Nevertheless, the diagnostic sensitivity of somatostatin receptor scintigraphy can vary, ranging from 65% to 100%, contingent upon factors such as the tumour's origin, the density and type of somatostatin receptors (SSTRs) expressed on the tumour cell surface, and the size of the tumour itself [15].

PET/CT imaging utilizing somatostatin receptor tracers, such as 68 Gallium DOTA-TATE, 68 Gallium-DOTATOC, or 68 Gallium-DOTA-NOC, has emerged as a valuable and clinically effective approach for assessing NETs. These tracers exhibit a strong affinity for SSTR2 while binding to other relevant subtypes in certain tumour types. Studies have reported an overall sensitivity of 91.7% and specificity of 93.5% for detecting NETs in individuals with MEN syndromes [16]. Currently, the European Neuroendocrine Tumour Society (ENETS) recommends the utilization of 68 Gallium-labelled somatostatin receptor PET/CT for diagnostic purposes in patients with nonfamilial, rare functional NETs [17]. This imaging technique proves useful for both staging purposes and detecting early recurrences following surgical resection of NETs.

The treatment strategies for EAS encompass various approaches. Firstly, nonspecific treatment focuses on managing associated complications such as hypertension, hyperglycaemia, and hypokalaemia.

Secondly, specific treatments aim to alleviate symptoms resulting from excessive ACTH production. One specific approach involves the use of medications to reduce endogenous steroid production by the adrenal glands, such as metyrapone. Metyrapone inhibits the enzyme steroid 11ß-hydroxylase, leading to a decrease in the conversion of 11-deoxycortisol to the more potent glucocorticoid cortisol. Studies have demonstrated that metyrapone can effectively improve clinical, hormonal, and biochemical parameters in patients with EAS. Common side effects of metyrapone include transient hypoadrenalism and hirsutism. Other medications, such as ketoconazole (which inhibits cytochrome P450), aminoglutethimide, and somatostatin analogues, are also utilized. Another treatment option involves the use of slow-acting cortisol-lowering drugs such as mitotane and mifepristone.

Additionally, adrenal ablation, which can be achieved through mitotane therapy, embolization, or adrenalectomy, is considered a means to decrease endogenous steroid production [2].

## Conclusions

Ectopic ACTH secretion should always be considered in the diagnostic workup of young patients with CS. There is a small but growing body of literature describing the correlation between ectopic ACTH secretion and thymic NETs. These findings underscore the importance of thorough investigation in cases of unexplained ACTH-dependent CS, as identifying the underlying ectopic source can guide appropriate therapeutic interventions. Additionally, the possibility of a MEN1 syndrome should be considered in all patients with thymic NETs.
